# Poverty-related diseases (PRDs): unravelling complexities in disease responses in Cameroon

**DOI:** 10.1186/s41182-016-0042-5

**Published:** 2017-01-14

**Authors:** Valerie Makoge, Harro Maat, Lenneke Vaandrager, Maria Koelen

**Affiliations:** 1 Health and Society (HSO) group, Wageningen University, P.O. Box 8130, , 6700 EW Wageningen, The Netherlands; 2 Knowledge Technology and Innovation (KTI) group, Wageningen University, Hollandseweg 1, 6708 KN Wageningen, The Netherlands; 3 Institute of Medical Research and Medicinal Plant studies (IMPM), P.O. Box 13033, Yaoundé, Cameroon

**Keywords:** Poverty-related diseases, Disease responses, Health belief model, Malaria, University students, Cameroon

## Abstract

**Background:**

In Cameroon, poverty-related diseases (PRDs) are a major public health concern. Research and policies addressing PRDs are based on a particular understanding of the interaction between poverty and disease, usually an association between poverty indicators and health indicators for a specific country or region. Such indicators are useful but fail to explain the nature of the linkages between poverty and disease or poverty and health. This paper presents results of a study among university students, unravelling how they perceive diseases, the linkages with poverty, their responses to diseases and the motivations behind reported responses.

Based on the health belief model, this cross-sectional study was carried out among 272 students at the universities of Buea and Yaoundé in Cameroon. Data were collected using questionnaires containing items matching the research objectives. The questionnaires were self-completed.

**Results:**

Malaria was considered as the most common disease perceived and also a major PRD. Contrary to official rankings of HIV/AIDS and TB, cholera and diarrhoea were considered as other major PRDs. Also, typhoid fever was perceived to be more common and a PRD than HIV/AIDS and TB combined. The most prominently attributed cause for disease was (lack of) hygiene. In response, students deployed formal and/or informal healthcare strategies, depending on factors like available money, perceived severity of the disease and disease type. Discrepancies were observed in respondents’ response to diseases generally and to malaria in particular. Even though, overall, respondents pre-dominantly reported a formal healthcare response toward diseases in general, for malaria, informal responses dominated. There was an overall strong awareness and (pro)activity among students for dealing with diseases.

**Conclusions:**

Although the high use of informal facilities and medication for malaria may well be a reason why eradication is problematic, this seems to be a deliberate strategy linked to an awareness of the limitations of the formal health system. In any intervention intended to foster health, it is therefore vital to consider people’s perceptions toward diseases and their response strategies. Our results give important leads to health promotion interventions to develop group-specific programs.

## Background

The eventual success of health-promoting interventions for infectious diseases in developing countries depends on a prior understanding of the complexities surrounding people’s efforts to respond to illnesses. Poverty makes such efforts more complicated. The recognition of linkages between poverty and health has led to the term poverty-related diseases (PRDs). PRDs are diseases whereby poverty is a factor that increases the chances of getting the disease and hinders proper treatment and cure [1, 2]. According to the World Health Organization (WHO), the major PRDs are malaria, human immuno-deficiency virus (HIV)/AIDS and tuberculosis [3]. Cameroon, located in Central Africa, is one of those nations in which PRDs are a significant public health concern. In Cameroon, for instance, everyone is at risk of malaria but the burden is felt more by the poor. Malaria accounts for most hospitalisations, and up to 40% of family income is reportedly spent on its prevention and treatment [4]. The prevalence of HIV in Cameroon is the highest in the sub-region of West and Central Africa, standing at 5.1% [5]. Also, about 33,000 tuberculosis infections are recorded each year, with over 2000 annual deaths [6] mostly in association with HIV [7, 8].

Research and policies addressing PRDs are based on a particular understanding of the interaction between poverty and disease, measured as an association between poverty indicators and health indicators for a specific country or region. Such indicators provide an adequate picture of the diseases most prevalent among the poor but do not further explain the nature of the linkages between poverty and disease or poverty and health [1, 9]. Besides disease prevalence and income levels, living conditions influence vulnerability towards certain diseases and are thus also important for understanding PRDs [10].

Rather than approaching PRDs from statistics, we study people’s own perspective on the connections between poverty and health. We investigated perceptions and responses to health issues among university students in Cameroon, a group that is well-educated but has an income level that on average is close to the official poverty line of US$1.25 a day (see Table 1). Common diseases in this group are similar to those in other groups in Cameroon, although some studies have reported a high prevalence of sexually transmitted infections (STIs) among students (e.g. 31% for gonorrhoea) due to a reduced awareness of preventive strategies [11]. Malaria has been reported to account for up to 65% of absences from schools [4, 12]. Many factors influence a person’s selective response to illness. The options available to acquire treatment or medication in Cameroon include government hospitals and healthcare centres, church-related hospitals and clinics, private doctors, private pharmacies (big and small), community health workers and street vendors [13]. The use of informal healthcare providers in Cameroon is common [14, 15]. Acquiring care from formal healthcare services is not always an obvious first step in Cameroon where most healthcare is an out-of-pocket expense [16, 17].

**Table 1:** Background characteristics of student respondents from UB and UNIYAO

		UB (*N* = 161)	UNIYAO (*N* = 111)	*p* value
		%	%	
Sex	Male	43.5	32.4	0.66
	Female	56.5	67.6	
Participants’ age in ranges	<25	77.4	52.3	<0.05
	25 or older	22.6	47.7	
Single people (vs married)	Married	0.6	1.8	0.363
	Single	99.4	98.2	
Participants’ income level (per month)	<20,000 FCFA	42.5	44.0	0.820
	20–50,000 FCFA	36.6	39.4	
	50–100,000 FCFA	11.8	10.1	
	>100,000 FCFA	9.2	6.4	

The aim of our study was to get a deeper understanding of the complexities surrounding responses towards diseases among university students in Cameroon. Such understanding is essential for the design and assessment of interventions geared at promoting health [18, 19] among poor groups in society.

**Fig. 1:**
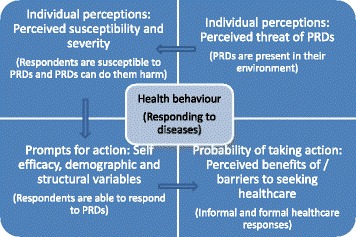
Operationalisation of disease responses in the health belief model

We used the health belief model (HBM) [20] to understand students’ response to diseases. The health belief model is a model commonly used in health education and health promotion that aims to predict and understand health behaviours of people in terms of belief patterns they may have [20–23]. According to the HBM, people’s response to diseases is guided by two main beliefs: a belief in a health threat (presence of disease) and a belief in the effectiveness of deploying a response strategy [20, 22]. Because PRDs are primarily infectious diseases, the first belief implies a perceived susceptibility to infection and a perception that this infection could be harmful. The second belief in the context of our study implies a belief that both formal and informal health facilities and medication can be used to respond to diseases and that these actions will be effective [22]. Figure 1 shows how we operationalise disease responses in the HBM.

The specific objectives of our study were to assess the perceptions of university students in Cameroon about common diseases and diseases related to poverty, to find out how they respond to diseases, to ascertain the motivations for their responses and finally, to identify the determinants (perceived benefits and barriers) for the participants’ use of the healthcare services.

## Methods

### Survey design and respondents

Our study was part of a larger project that had as objective to create an understanding of the social and material dynamics that enable the capacity to preserve health, anticipate health risks and mitigate or recover from stressors such as PRDs in Cameroon. This study took place between 2013 and 2014 and was carried out in the Southwest and Centre regions of Cameroon.

Respondents were selected among students at the University of Buea (UB), located in the Southwest region, 4.1537° N, 9.2920° E, and the University of Yaoundé (UNIYAO), located in the Centre region 3.8574° N, 11.5014° E of Cameroon. UB, founded in 1995, has a population of about 12,000 students [24]. UNIYAO, the oldest university in Cameroon, was founded in 1962 and has a population of about 33,000 students [25]. Every year, over 1000 students seek entry into the universities of Buea or Yaoundé. Potential students originate from different regions of Cameroon and have either an anglophone or a francophone culture and settle into these settings for studies. Entry into UB is on a competitive basis following results of the general certificate examination (GCE A’L), whereas entry into UNIYAO is not based on competition. Both universities are state-owned and are top-ranked universities in Cameroon. University students were selected for this study because, despite having education and career opportunities that are better than for most people, during their study, most students live in deprived conditions and face financial challenges. Their experiences and disease response strategies are important for understanding the complexities that exist between poverty and health.

University students reside in what we refer to as campus settings. Most university students live in rented rooms in neighbourhoods close to the university because on-campus rooms are too few to cater for the high demand. Student residences vary in size and can have as few as five rooms, whereas others have up to 40 rooms. For our study, the neighbourhoods visited were the Ngoa-Ekelle in Yaoundé and the Molyko in Buea situated within a few hundred metres of the university and each housing hundreds of students. These neighbourhoods are quite similar, both bustling with commercial activities that centre around students’ needs such as photocopying and printing stalls, small restaurants, bars, small pharmacies and cyber cafés. Respondents were following various study programmes offered by the university. The universities offer no free healthcare services to the students, who therefore have to seek healthcare off-campus at personal expense.

### Survey instrument

From initial observations and preliminary conversations with students, we obtained an image of the key issues and conditions affecting students’ health, from which we designed a questionnaire in line with the health belief model [20]. The questionnaire was comprised of closed questions with yes/no answer options and questions with pre-defined answer categories, with the possibility for multiple responses in some questions.

### Individual perceptions

The construct of a perceived threat of disease is characterised by a perceived vulnerability and a perceived severity of disease. These factors have been shown to influence how people respond to diseases [20, 22]. Perceiving diseases as a threat means recognising their presence and their ability to affect a person. This factor was included to pinpoint specific diseases affecting the health and well-being of university students and was measured by asking students to identify what diseases they perceive as common and what diseases they associate with poverty. In addition to the WHO-listed top three PRDs, other diseases such as cholera, diarrhoea, typhoid fever and meningitis were included in this question.

Perceived vulnerability was constructed as the perception of the risk of contracting diseases. Questions focused on factors reported in the literature as having a direct impact on health and increasing vulnerability to diseases, such as food availability, balanced diet, good and permanent water, and sanitation challenges [26, 27].

Perceived severity, which implies the perception that PRDs could cause harm to respondents, was not specified in the questionnaire because other studies have established this aspect with regard to malaria and HIV in Cameroon [4, 28–30].

### Prompts to action

A prompt to action refers to the recognition that actions can be taken to respond to diseases faced by university students. Questions asked included “How would you normally respond to diseases in general?” and “How would you normally respond to malaria?” The additional question about malaria gives further insights into students’ responses to PRDs. Because prevalence, morbidity and mortality due to malaria are high in Cameroon [31, 32], all students are likely to have experience in dealing with it.

### Probability of taking action

Converting prompts to action into real action depends on the perceived benefits and barriers relating to the effectiveness of the intended action [20]. We included several options for action in the questionnaire, covering both formal and informal medical facilities. An example of a question asked is “What determines whether you seek formal or hospital healthcare?”

Socio-demographic variables like age, sex and other variables shown to influence how people respond to diseases [33] were also obtained in this study.

### Procedure

The questionnaires were pre-tested among students at the Catholic University in the Southwest region to ensure that the questions were understandable. Consequent to the pre-test, minor amendments were made relating to the comprehensibility of the questions.

The questionnaires were self-administered. The respondent sampling method followed that previously described by Moyou-Somo et al. [32] whereby, for a population of less than 1000 inhabitants, one out of every two houses is selected for the research. The first building selected was the one closest to the main road, and from this, we moved inwards into the neighbourhood. In our study, we considered the student’s room as a house. The first author and trained assistants went into each student building, explained the study and requested participation. The questionnaires were left with the students and picked up later in the day. In total, 300 questionnaires were distributed, and 272 respondents completed and returned the questionnaire, giving a response rate of 90%.

### Data analysis

The data were analysed using SPSS Statistics version 22 (SPSS Inc. IBM). Before analysis, data from pre-coded questionnaires were entered into SPSS and checked for errors by the first author and an assistant. Chi-square and ANOVA tests were used to analyse differences between the universities as well as in the factors included in the survey, such as response strategies towards diseases in general and towards malaria in particular. We performed logistic regression analysis to explain trends seen in people’s response to malaria. Included in the model were socio-demographic factors (age, sex and income). We also calculated the odds ratio of factors shown to have an influence on people’s health, such as food, water and sanitation challenges experienced by people and seeking formal healthcare for malaria; *p* values <0.05 were considered to be significant.

Ethical clearance for this study was provided by the Wageningen University review board. The aim of the study was carefully explained to all respondents. Respondents signed an informed consent form before participating in the study.

## Results

### Respondents’ characteristics

As shown in Table 1, 272 students (39% males and 61% females) participated in the survey, 98.9% of whom were single. The income of most university students was less than 20,000 FCFA (less than US$34.7) per month. Respondents from the two universities showed no significant differences in background variables such as sex, marital status and income. However, the mean age of students at UB was slightly lower than that of students at UNIYAO (23 and 24.5, respectively; *F* (1,264), *p* < 0.001). Further analysis revealed that age did not play a significant role in respondents’ response to diseases; therefore, all respondents are treated as one group.

### Respondents’ belief in diseases as a threat and perceived vulnerability towards PRDs

The three diseases most commonly identified by respondents were malaria (91.1%), typhoid (35.7%) and HIV/AIDS (16%). The three major PRDs perceived were malaria, cholera and diarrhoea. WHO-listed PRDs, such as HIV/AIDS and tuberculosis (TB), had lower ratings (Table 2).

**Table 2:** Respondents’ classification of common diseases and PRDs

Diseases	Respondents’ classification (%)^a^	
	Common diseases	PRDs
Malaria	91.1	36.4
Cholera	6.7	34.4
Diarrhoea	13.8	31.2
Typhoid fever	35.7	28.1
HIV/AIDS	16	20.2
STIs	10.8	9.9
TB	2.6	5.9
Meningitis	1.5	na

*na* not asked

^a^More than one response was possible

The presence of diseases was mostly attributed to (lack of) hygiene (53.2%). Poor hygiene conditions increase respondents’ vulnerability to diseases. For example, many respondents had toilets in their rooms for personal use (61.7%), but another group used pit toilets that they shared with neighbours, with a consequent increased infection risk. Other attributed causes were poverty (31.3%), climatic conditions (29.8%) and lack of knowledge (23.8%).

### Prompts to action: disease responses

Table 3 shows a picture of the different ways in which students respond to diseases relative to socio-demographic characteristics. It can be seen that, irrespective of socio-demographic characteristics, most people reported that they would use the formal healthcare facilities as their general response to health challenges.

**Table 3:** Variation of health-seeking practices in relation to socio-demographic differences

Socio-demographic variables	Response to diseases in general (*N* = 272)			
		Formal %	Informal %	Both %
Sex	Male	71.1	23.7	5.2
	Female	70.0	23.8	6.3
Participants’ age in ranges	<25	73.1	22.2	4.8
	25 or older	65.5	26.2	8.3
Marital status	Married	100	0.0	0.0
	Single	70.4	24.1	5.5
Participants’ income level in thousand FCFA	<20	66.7	30.5	2.9
	20–50	75.0	17.7	7.3
	50–100	65.4	26.9	7.7
	>100	71.4	19.0	9.5

*Note*: Figures may not add up to exactly 100% because of rounded values

### Response to malaria

The picture of using the formal healthcare system as a general response to diseases (70.4%) changes for malaria. Table 4 shows these discrepancies. In the case of malaria, the use of informal treatment and medication was the dominant response (86%), with only about 6% of respondents reporting that they would employ formal healthcare services.

**Table 4:** Discrepancies in disease responses towards malaria and other diseases

	Formal %	Informal %	Both %
Health-seeking practices in general	70.4	23.7	5.8
Health-seeking practices in the case of malaria	6.4	86.1	7.5

In an effort to explain these discrepancies, we investigated predictors of seeking formal healthcare as well as the odds of water, sanitation and food challenges influencing the way people respond to malaria. Table 5 provides descriptive information regarding the presence or absence of challenges and the selected response to malaria. Logistic analysis regression (results shown in Table 6) did not reveal any socio-demographic factors to be predictors of seeking formal healthcare for malaria. Also, none of those experiencing sanitation, food and water challenges showed increased or decreased odds of seeking formal healthcare in the case of malaria attacks (see Table 7).

**Table 5:** Variation in sanitation, food and water challenges with respect to malaria responses

Sanitation, food and water challenges		Response to malaria		
		Formal %	Informal %	Both %
Does participant share toilet with other houses?	Yes	3.8	33.2	3.8
	No	2.6	52.3	3.8
Are there water cuts in the neighbourhood?	Yes	5.7	54.0	5.7
	No	0.8	32.1	1.9
Does participant eat or drink herbs and other parts of plants to prevent or cure disease?	Yes	6.0	43.4	7.2
	No	0.4	42.6	0.4
Is food readily available for the participant?	Yes	3.4	35.7	3.8
	No/sometimes	3.0	50.4	3.8
Does participant cook his/her meals?	Yes/sometimes	5.3	74.8	6.8
	No	1.1	11.3	0.8
Does participant consider his/her diet to be balanced?	Yes/sometimes	3.0	49.1	5.2
	No	3.4	37.1	2.2
Are there days when participant misses one or more meals?	Yes	6.4	77.4	6.8
	No	–	8.6	0.8

*Note*: Values on table may not add up to exactly 100% because of rounded values

**Table 6:** Logistic regression model with seeking formal healthcare in the event of malaria as dependent variable

Variables	*B*	S.E.	Sig.	Exp(*B*)
Age	0.098	0.075	0.191	1.103
Sex	−0.614	0.533	0.249	0.541
Income	−0.161	0.311	0.604	0.851
Nagelkerke R2				0.041

**Table 7:** Odds ratios and 95% confidence interval for seeking formal healthcare in the presence of threats

Predictor variables (threats)	OR (95% CI)	*p* value
Toilet sharing	1.076 (0.616–1.879)	0.798
Water cuts	0.818 (0.456–1.466)	0.499
Drink/eat herbs	0.591 (0.334–1.043)	0.068
Food availability	1.135 (0.651–1.979)	0.655
Food cooking	1.714 (0.795–3.699)	0.166
Balanced meal	1.399 (0.808–2.421)	0.230
Food miss day	0.873 (0.334–2.284)	0.782

### Top informal healthcare responses towards malaria

Using small pharmacies (any small shop selling some medication) were reportedly the most common way in which respondents dealt with malaria (52.2%). Self-medication practices (20.6%) were also commonly used. Medicinal plants, usually a combination of different plants, were used for self-medication, with 56.7% of respondents reportedly using a combination of plants to prevent or treat malaria. Common medicinal plants used were fever grass, pawpaw leaves, aloe vera, mango and guava leaves. Using street vendors as informal healthcare providers was the third most commonly reported practice (18.8%).

### Probability of taking action: determinants for seeking informal and formal healthcare

Formal healthcare was often sought after self-medication failed (33.2%) or when illness was severe (22.9%). Having money was the most important factor enabling people to make use of official medical facilities. Other important factors were perceived severity of the disease and duration of the illness (Table 8).

**Table 8:** Determinants in favour of using formal healthcare facilities

Determinants	% (*N*)
Money	53.9 (144)
Severity of illness	40.8 (109)
Duration of illness	20.2 (54)
Fear	7.5 (20)
Unavailability of drugs	6.7 (18)
Attitude of hospital staff	4.1 (11)
Time	2.6 (7)
Distance to healthcare service	1.5 (4)

*Note*: Multiple responses were possible

## Discussion

The aim of this study was to get a deeper understanding of the complexities regarding the way in which university students respond to diseases present in their environment. Before we delved into how students respond to PRDs, it was important to identify what students consider to be PRDs. Our results show that university students perceive malaria as the top common disease and PRD. Typhoid fever is considered a common disease but was not ranked highly as a PRD, whereas cholera, which was not perceived as very common, was classified as a major PRD. Although our study does not provide a clear explanation for this ranking, it is likely that respondents’ ideas of interconnections between hygiene, health and extreme poverty carry a symbolic meaning of the kind of situation they strongly reject [34]. In other words, cholera, and the scare it generates, represents poor social well-being culminating in poor health. This suggests that people’s capacity to maintain personal hygiene and ensuring a liveable environment are two sides of the same coin. The fight against PRDs therefore should focus strongly on facilitating good hygiene practices. Overall, official health authorities and international agencies may underestimate people’s perception of health and hygiene and their capacity to adapt to changing circumstances such as outbreaks of infectious diseases [35]. We show this in our study by highlighting people’s role in responding to diseases that affect them and by underlining the importance of taking this response into account in the design of interventions whose goal is to foster health.

It is noteworthy that respondents’ classification of major PRDs differed from that used by health bodies such as the United Nations, WHO, the European Commission [36] and Cameroon’s Ministry of Public Health [5]. The United Nations, for instance, listed the major PRDs as malaria, HIV/AIDS and TB in millennium development goal 6 to be eradicated in an allocated 15-year time span. This was not achieved and these diseases are now included in the third sustainable development goal (SDG 3), to be achieved by 2030 [37]. Emphasis on achieving such goals highlights the importance of problems faced by people living in conditions of poverty. Studies such as the one presented here provide insights into the complexities of the interactions between poverty and health—insights that are useful for both governmental and non-governmental organisations and international health bodies in the formulation of policy to improve people’s health and well-being and provide information needed for the design and implementation of adequate interventions to promote human health.

As expected, the classification of malaria as a major PRD and a public health concern was uncontested by our respondents, and this is confirmed by other studies in Cameroon [31, 38], Nigeria [39], Ethiopia [40], Kenya [41] and Uganda [42]. The other two WHO-listed PRDs differed in importance in our respondents’ perspective. HIV/AIDS was perceived as the third most common disease by 16% of respondents, but it was not ranked among the top three PRDs. This is probably because students are at an age and life stage in which they take a more adventurous and active approach to sexuality [43–45] and therefore may not necessarily link HIV/AIDS to poverty. Our study showed that less than 3% of respondents perceived TB as being common and only about 6% perceived it as a PRD. Actually, in the list of diseases given to respondents, TB appeared at the bottom end of both rankings. Apparently, students do not consider TB a common disease and do not associate it with poverty. Typhoid fever on the other hand was perceived as more common and also more a PRD than HIV/AIDS and TB combined (see Table 2). Our results reveal a chasm between world health bodies’ official classifications and what people in conditions of poverty themselves perceive. Although people’s perceptions are not infallible, health programmes are likely to be more effective if they match people’s perception of disease risk with options for improving their health. We suggest that disease risks perceived by people living in conditions of poverty should be the focus of health-promoting agencies’ in the fight against PRDs. Further research could look into other groups in society as well and develop in-country differentiated health programmes.

By looking at PRDs from the respondents’ viewpoint, we show that PRDs are not only a list of diseases but also entail people’s perceptions of the interlinkages between poverty and health, a factor that we consider a relevant focus for the successful achievement of SDG 3.

By identifying common diseases and PRDs through the respondents’ eyes, we reveal an interesting perspective on the major threats to students’ health and well-being. For one thing, we ascertain factors that increase students’ vulnerability to diseases. Some of these factors are under the students’ control and others are not. On the one hand, poor hygiene conditions were perceived by respondents as the most pronounced reason for disease presence. This was rated even higher than poverty, even though both may be interlinked [1]. These conditions can be partly controlled by the students themselves, when they clean up their own surroundings to partially reduce the risk of malaria infection [46]. On the other hand, other factors, for example poor housing conditions as observed in the neighbourhoods, are beyond the students’ control as they have to take what is available due to the shortage of accommodation possibilities.

Our respondents also attributed disease presence to climatic conditions, lack of knowledge and poor education. Other studies carried out in UB confirmed, for example, that the high prevalence of STIs was linked to students’ lack of knowledge on ways to protect themselves [11]. This underlines the need for group-specific health education interventions.

Moreover, our study revealed that the way students respond to disease was not always clear-cut; rather, it was influenced by disease genre. The response to malaria differed from the response to diseases in general. The general response to diseases was indicated as being mostly through formal healthcare facilities (70%). Money was reported as an important indicator for deploying this response strategy. However, our results show different strategies in spending scarce money. Even when the costs of hospital consultation, laboratory examination and buying medication from hospitals can be covered, the decision to do so can be postponed, depending on other factors, most prominently the perceived severity of the disease. Other studies have reported a similar strategic use of available resources [47–51]. Perceived severity is an obvious incentive for hospital-based action [22]. However, confounding factors could be the pain caused by the condition, duration of the illness, unavailability of medication at home, the failure of self-medication to alleviate symptoms or fear instilled by the disease condition. These factors stand on the one hand as enablers of hospital-based responses and, on the other hand, as barriers to this route. Furthermore, these factors indicate the other processes that could occur between the onset of symptoms and the use of formal medical facilities.

Interestingly, our study showed that, in the case of malaria, students’ response strategy was very different from that promulgated by health agencies and medical experts. Most respondents (86%) reported using informal health services and medicine as a response to malaria. This indicates a belief that the informal health sector is beneficial and effective in the case of malaria. It is noteworthy, though, that malaria is well-perceived as a severe disease in Cameroon [30, 52] and that our respondents indicated that one of the determinants for seeking formal healthcare was the perceived severity of the disease. Students’ response to malaria would therefore be expected to align with what they said was their general response to diseases. However, this was not the case. People obviously do not display a clear-cut logical pattern of response whereby they treat all diseases in the same way. Other factors may play a role in their decisions. These could be psychological or financial. Psychologically, perceptions are important. High-perceived severity of disease, for example, is a reason reported for hospital care [53]. Financial factors too were indicated. In the case of malaria, respondents opted to use small pharmacies, self-medication practices (with medicinal plants) and street vendors. It is not clear from our study why informal healthcare options are chosen to respond to malaria. A possible explanation could be that malaria is very common [4, 28] and an integral part of everyday life. Its constant presence may reduce the perception that it is a life-threatening disease and numb people to its deleterious effects. A qualitative study among a sample of respondents living in these same settings under similar conditions indicated that proximity and money-saving characteristics of informal healthcare providers such as small pharmacies and street vendors made them more enticing options [54]. This has also been reported in other studies [55]. It should be noted that the reason for informal healthcare in the case of malaria could not be explained by socio-demographic characteristics, and the odds did not increase or decrease with challenges that people experienced in relation to sanitation, food and water.

The insights revealed in our study are worthy of consideration in the context of health promotion interventions, as they could throw more light on why malaria is persistent and still a number one problem in Cameroon just like in other parts of sub-Saharan Africa. For instance, our respondents have shown strong awareness and (pro)activity in relation to malaria, but this may well be a reason why eradication is problematic. Aspects such as (1) self-diagnoses of malaria by people leading to (2) (im)proper self-medication practices without necessarily following treatment recommendations or appropriate treatment may have unfavourable outcomes. Also, a link may exist between using the informal sector (small pharmacies, street vendors) and the persistence of malaria, as well as the development of resistance to antimalarial drugs. Our findings therefore underline the need for integrated and all-encompassing strategies in the fight against malaria.

### Study limitations

Our study was cross-sectional in nature. A cross-sectional study refers to a study in which data are collected from respondents at a specific point in time and over a short period. In this way, a cross-sectional study will give a picture of the study of interest at the time it is carried out [56]. Our study therefore may not have unravelled all the complexities that change with time in the settings. Even though the perceived severity of malaria is already established in Cameroon, it would have been interesting to see how respondents perceive the severity of all WHO-ranked major PRDs as well, especially TB, which they did not perceive as a major PRD. By using the health belief model as a framework for our study, we may have omitted some aspects that also play a role in the way people respond to disease, such as the role of significant others in the decision to respond to diseases in particular ways.

## Conclusions

The aim of this study was to acquire a deeper understanding of the interaction between poverty and disease by investigating the health beliefs of university students in Cameroon. Our study showed that students are well aware of what constitutes common diseases and also display strong beliefs about what diseases can be attributed to poverty. Contrary to official rankings, students consider malaria, cholera, diarrhoea and typhoid fever as major PRDs. Moreover, the results revealed that students consider (lack of) hygiene as a more prominent cause of disease than poverty. Respondents also displayed strong beliefs about their capacity to respond to diseases. Our study found that they deploy both formal and informal response strategies towards diseases, depending on factors like having money to afford the services, perceived severity of the disease, disease genre or belief in the effectiveness of an action to bring relief from the burdens imposed by diseases. A remarkable finding of our study was that university students adopt a mostly informal response to malaria. A better understanding of the practices, surrounding beliefs and group-specific responses to malaria is essential for the effective management and control of the disease.

## Abbreviations

HBM: Health belief model
HIV: Human immuno-deficiency virus
PRD: Poverty-related disease
SDG: Sustainable development goal
STIs: Sexually transmitted infections
TB: Tuberculosis
UB: University of Buea
UNIYAO: University of Yaounde
WHO: World Health Organization

## References

[1] Stevens P (2004). Diseases of poverty and the 10/90 gap.

[2] Singh AR, Singh SA (2008). Diseases of poverty and lifestyle, well-being and human development. Mens Sana Monographs.

[3] WHO. Global report for research on infectious diseases of poverty. World Health Organization. 2012. http://apps.who.int/iris/bitstream/10665/44850/1/9789241564489_eng.pdf. Accessed 17 Sept 2015.

[4] CCAM. About_malaria: for a malaria free Cameroon. A bilingual publication of the Cameroon Coalition Against Malaria. 2009;2(1):1–28

[5] MINSANTE (2009). Profil des Estimations et Projections en Matière de VIH et SIDA au Cameroun 2010–2020.

[6] WHO (2011). Towards universal access to diagnosis and treatment of multidrug-resistant and extensively drug-resistant tuberculosis by 2015: WHO progress report 2011.

[7] Cambanis A, Ramsay A, Yassin MA, Cuevas LE (2007). Duration and associated factors of patient delay during tuberculosis screening in rural Cameroon. Trop Med Int Health.

[8] Pefura-Yone EW, Kengne AP, Kuaban C (2014). Non-conversion of sputum culture among patients with smear positive pulmonary tuberculosis in Cameroon: a prospective cohort study. BMC Infect Dis.

[9] Von Philipsborn P, Steinbeis F, Bender ME, Regmi S, Tinnemann P. Poverty-related and neglected diseases—an economic and epidemiological analysis of poverty relatedness and neglect in research and development. Glob Health Action. 2015;8. doi: http://dx.doi.org/10.3402/gha.v8.25818. 10.3402/gha.v8.25818 PMC4306754 25623607

[10] WHO. Housing and health. 2010. http://www.who.int/hia/housing/en/. Accessed 12 Sept 2016.

[11] Nkuo-Akenji T, Nkwesheu A, Nyasa R, Tallah E, Ndip R, Angwafo IF (2007). Knowledge of HIV/AIDS, sexual behaviour and prevalence of sexually transmitted infections among female students of the University of Buea, Cameroon. Afr J AIDS Res.

[12] DHS. The DHS program—Cameroon: standard DHS, 2011. Demographic Health Survey. Cameroon: Institut Nationale de la Statistique; 2011. pp. 188–204.

[13] Kamgnia B (2006). Use of health care services in Cameroon. Int J Appl Econom Quant Stud.

[14] Hughes R, Chandler C, Mangham-Jefferies L, Mbacham W. Medicine sellers’ perspectives on their role in providing health care in North-West Cameroon: a qualitative study. Health Pol Plan. 2013;doi: 10.1093/heapol/czs103. 10.1093/heapol/czs103 PMC3753882 23197432

[15] Crabbe F, Carsauw H, Buve A, Laga M, Tchupo J, Trebucq A (1996). Why do men with urethritis in Cameroon prefer to seek care in the informal health sector?. Genitourin Med.

[16] Jaja PT. Health-seeking behaviour of Port Harcourt city residents: a comparison between the upper and lower socio-economic classes. J Public Health Africa. 2013; doi: http://dx.doi.org/10.4081/jphia.2013.e9. 10.4081/jphia.2013.e9 PMC5345428 28299098

[17] Kankeu HT, Ventelou B (2016). Socioeconomic inequalities in informal payments for health care: an assessment of the ‘Robin Hood’ hypothesis in 33 African countries. Soc Sci Med.

[18] Mackian S, Bedri N, Lovel H (2004). Up the garden path and over the edge: where might health-seeking behaviour take us?. Health Pol Plan.

[19] Grundy J, Annear P (2010). Health-seeking behaviour studies: a literature review of study design and methods with a focus on Cambodia.

[20] Janz NK, Becker MH (1984). The health belief model: a decade later. Health Educ Behav.

[21] Hochbaum G, Rosenstock I, Kegels S. Health belief model. Washington: United States Public Health Service; 1952.

[22] Koelen MA, van den Ban AW (2004). Health education and health promotion.

[23] Green EC, Murphy E (2014). Health belief model. The Wiley Blackwell encyclopedia of health, illness, behavior, and society.

[24] University of Buea. About UB: University of Buea. 2014. http://www.ubuea.cm/about/. Accessed 15 Aug 2015.

[25] Institut National de la Statistique (2010). Annuaire Statistique du Cameroun.

[26] WHO (2004). Guidelines for drinking-water quality: recommendations.

[27] Mensah P, Yeboah-Manu D, Owusu-Darko K, Ablordey A (2002). Street foods in Accra, Ghana: how safe are they?. Bull World Health Organ.

[28] MINSANTE (2007). Plan Strategique Nationale de la lutte contre le paludisme au Cameroon 2007–2010.

[29] Makoge V, Maat H, Edward N, Emery J (2016). Knowledge, attitudes and practices towards malaria in Mbonge and Kumba sub-divisions in Cameroon. Int J Trop Dis Health.

[30] Kimbi HK, Nkesa SB, Ndamukong-Nyanga JL, Sumbele IU, Atashili J, Atanga MB (2014). Knowledge and perceptions towards malaria prevention among vulnerable groups in the Buea Health District, Cameroon. BMC Public Health.

[31] Ndo C, Menze-Djantio B, Antonio-Nkondjio C. Awareness, attitudes and prevention of malaria in the cities of Douala and Yaounde (Cameroon). Parasit Vectors. 2011; doi: 10.1186/1756-3305-4-181. 10.1186/1756-3305-4-181 PMC3192766 21933411

[32] Moyou-Somo R, Essomba P, Songue E, Tchoubou NN, Ntambo A, Hiol HN, et al. A public private partnership to fight against malaria along the Chad-Cameroon pipeline corridor: I. Baseline data on socio-anthropological aspects, knowledge, attitudes and practices of the population concerning malaria. BMC Public Health. 2013;13(1):1023. 10.1186/1471-2458-13-1023 PMC4228441 24168316

[33] Glanz K, Rimer BK, Viswanath K (2008). Health behavior and health education: theory, research, and practice.

[34] Douglas M (2002). Rules and meanings: the anthropology of everyday knowledge.

[35] Richards P (2016). Ebola: how a people’s science helped end an epidemic.

[36] Gryseels B, Zumla A, Troye-Blomberg M, Kieny MP, Quaglio G, Holtel A (2009). European Union conference on poverty-related diseases research. Lancet Infect Dis.

[37] Sachs JD (2012). From millennium development goals to sustainable development goals. Lancet.

[38] Nkuo Akenji TK, Ntonifor NN, Ching JK, Kimbi HK, Ndamukong KN, Anong DN (2005). Evaluating a malaria intervention strategy using knowledge, practices and coverage surveys in rural Bolifamba, southwest Cameroon. Trans R Soc Trop Med Hyg.

[39] Audu O, Bako Ara I, Abdullahi Umar A, Nanben Omole V, Avidime S. Sociodemographic correlates of choice of health care services in six rural communities in North Central Nigeria. Adv Public Health. 2014; http://dx.doi.org/10.1155/2014/651086.

[40] Jima D, Tesfaye G, Deressa W, Woyessa A, Kebede D, Alamirew D (2005). Baseline survey for the implementation of insecticide treated mosquito nets in Malaria control in Ethiopia. Ethiop J Health Dev.

[41] Imbahale SS, Fillinger U, Githeko A, Mukabana WR, Takken W (2010). An exploratory survey of malaria prevalence and people’s knowledge, attitudes and practices of mosquito larval source management for malaria control in western Kenya. Acta Trop.

[42] Musoke D, Karani G, Ssempebwa JC, Musoke MB. Integrated approach to malaria prevention at household level in rural communities in Uganda: experiences from a pilot project. Malar J. 2013; doi: 10.1186/1475-2875-12-327. 10.1186/1475-2875-12-327 PMC3848758 24041445

[43] Siegel DM, Klein DI, Roghmann KJ (1999). Sexual behavior, contraception, and risk among college students. J Adolesc Health.

[44] Atuyambe LM, Kibira SPS, Bukenya J, Muhumuza C, Apolot RR, Mulogo E. Understanding sexual and reproductive health needs of adolescents: evidence from a formative evaluation in Wakiso district, Uganda Adolescent Health. Reprod Health. 2015; doi: 10.1186/s12978-015-0026-7. 10.1186/s12978-015-0026-7 PMC4416389 25896066

[45] Sekirime WK, Tamale J, Lule JC, Wabwire-Mangen F (2001). Knowledge, attitude and practice about sexually transmitted diseases among university students in Kampala. Afr Health Sci.

[46] WHO. World malaria report 2013. http://www.who.int/malaria/publications/world_malaria_report_2013/wmr2013_no_profiles.pdf?ua=1. Accessed 3 Sept 2015.

[47] Adedokun BO, Morhason-Bello IO, Ojengbede OA, Okonkwo NS, Kolade C (2012). Help-seeking behavior among women currently leaking urine in Nigeria: is it any different from the rest of the world?. Patient Prefer Adher.

[48] Abdulraheem IS, Parakoyi DB (2009). Factors affecting mothers’ healthcare‐seeking behaviour for childhood illnesses in a rural Nigerian setting. Early Child Dev Care.

[49] Burtscher D, Burza S. Health-seeking behaviour and community perceptions of childhood undernutrition and a community management of acute malnutrition (CMAM) programme in rural Bihar, India: a qualitative study. Public Health Nutr. 2015; doi: 10.1017/S1368980015000440. 10.1017/S1368980015000440 PMC10271539 25753193

[50] Ahmed SM, Adams AM, Chowdhury M, Bhuiya A (2003). Changing health-seeking behaviour in Matlab, Bangladesh: do development interventions matter?. Health Pol Plan.

[51] Wang Q, Brenner S, Leppert G, Banda TH, Kalmus O, De Allegri M (2015). Health seeking behaviour and the related household out-of-pocket expenditure for chronic non-communicable diseases in rural Malawi. Health Pol Plan.

[52] Sumbele IUN, Samje M, Nkuo-Akenji T (2013). A longitudinal study on anaemia in children with Plasmodium falciparum infection in the Mount Cameroon region: prevalence, risk factors and perceptions by caregivers. BMC Infect Dis.

[53] Biswas P, Kabir ZN, Nilsson J, Zaman S (2006). Dynamics of health care seeking behaviour of elderly people in rural Bangladesh. Inter J Ageing Later Life.

[54] Makoge V, Maat H, Vaandrager L, Koelen M. Health seeking behaviour towards poverty-related diseases (PRDs): a qualitative study of people living in camps and on campuses in Cameroon. PLoS Negl Trop Dis. 2017;11(1): e0005218. doi:10.1371/journal.pntd.0005218. 10.1371/journal.pntd.0005218 PMC5214973 28052068

[55] Ondicho JM. Factors associated with use of herbal medicine among the residents of Gucha sub-county, Kenya. Thesis for Master of Science in Applied Epidemiology, Jomo Kenyatta University of Agriculture and Technology, Juja, Kenya; 2015.

[56] Levin KA (2006). Study design III: cross-sectional studies. Evid Based Dent.

